# Reviving Hope: Overcoming Deep Brain Stimulation Failure With Partial Cranial Nerve Radiofrequency Ablation in Primary Meige Syndrome

**DOI:** 10.1002/brb3.71383

**Published:** 2026-04-14

**Authors:** Xue Li, Min Yan, Bing Huang, Lina Yu, Huabo Liu, Hao Huang, Ming Yao

**Affiliations:** ^1^ Pain Management Center, Department of Anesthesiology, the Second Affiliated Hospital Zhejiang University School of Medicine Hangzhou China; ^2^ Key Laboratory of the Diagnosis and Treatment of Severe Trauma and Burn of Zhejiang Province Hangzhou China; ^3^ Zhejiang Key Laboratory of Pain Perception and Neuromodulation Hangzhou China; ^4^ Department of Pain Medicine The Affiliated Hospital of Jiaxing University Jiaxing China; ^5^ Department of Anesthesiology, the Second Affiliated Hospital Zhejiang University School of Medicine Hangzhou China; ^6^ Department of Anesthesiology Zhoushan Hospital of Zhejiang Province Zhoushan China

**Keywords:** cranial nerve, deep brain stimulation, primary Meige syndrome, radiofrequency ablation

## Abstract

**Introduction:**

Deep brain stimulation (DBS) is a standard treatment for refractory primary Meige syndrome (PMS), yet 30%–50% of patients experience suboptimal outcomes. Management strategies for these “DBS failures” remain undefined. This study evaluates the efficacy of partial cranial nerve radiofrequency ablation (RFA) as a salvage therapy for this challenging population.

**Methods:**

We report a retrospective series of three PMS patients classified as “DBS non‐responders” based on clinical refractory status or ineligibility for lead revision. Patients underwent imaging‐guided partial RFA of the facial nerve (for blepharospasm) and/or mandibular nerve (for oromandibular dystonia). The procedure utilized a graded thermal protocol, titrated strictly to a mild, controlled functional impairment clinical endpoints.

**Results:**

Immediate symptom remission was achieved in all cases. The Burke–Fahn–Marsden Dystonia Rating Scale (BFMDRS) Movement subscores decreased significantly (e.g., from 9 to 0 in Case 1, and 14 to 0.5 in Case 2). Procedural sequelae, including mild facial paralysis and masticatory weakness, were transient and resolved within 3–6 months. One patient experienced symptom recurrence at 14 months, which was successfully managed via repeat RFA.

**Conclusion:**

Partial cranial nerve RFA is a feasible, effective, and repeatable salvage therapy for PMS following DBS failure. By targeting the peripheral conduction pathway, it offers a distinct mechanism of action and a practical alternative when central neuromodulation proves insufficient. Future studies with larger cohorts are warranted to validate these preliminary findings.

## Graphical abstract

Partial cranial nerve radiofrequency ablation (RFA) represents a feasible and potentially effective salvage therapy for Primary Meige Syndrome following deep brain stimulation (DBS) failure. Targeting peripheral pathways may facilitate rapid symptom remission with manageable sequelae, offering a practical alternative when central neuromodulation proves insufficient.

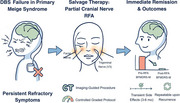

## 1 Introduction

Primary Meige syndrome (PMS) is a segmental craniocervical dystonia characterized by involuntary, axisymmetric movement of craniocervical muscles (LeDoux 2009). Although the condition was first described over a century ago (Henry 1910), its management remains a significant clinical challenge due to its obscure pathogenesis and the lack of specific oral pharmacotherapies. While local botulinum toxin (BoNT) injections offer effective symptomatic relief (Jochim et al. 2020), the benefits are transient (typically 2–6 months). Furthermore, long‐term efficacy is often limited by potential adverse effects and the development of immunoresistance (Hassell and Charles 2020).

For refractory cases, deep brain stimulation (DBS) targeting the bilateral globus pallidus internus (GPi) or subthalamic nucleus (STN) has been established as a therapeutic option. However, clinical outcomes are variable, with only 50%–70% of patients achieving satisfactory relief (Hao et al. 2024; Xie et al. 2025). Moreover, DBS is a costly and technically demanding procedure requiring stereotactic neuro‐navigation and craniotomy (Xie et al. 2025). Given the invasive nature and high cost of implantation, treatment failure can be psychologically devastating for patients who harbor high expectations for recovery. Consequently, the management of PMS patients who are non‐responders to DBS represents a critical therapeutic dilemma.

This article reports three cases in which partial radiofrequency ablation (RFA) of the extracranial branches of the involved cranial nerves was successfully employed as a salvage therapy following DBS failure, providing potential insights for the management of this difficult‐to‐treat population.

## 2 Materials and Methods

### 2.1 Study Design and Patient Selection

The study protocol was approved by the Human Research Ethics Committees of both participating centers (Certificate Nos. 2022‐LY317 and 2024–031). Written informed consent was obtained from all patients and their families after a detailed explanation of the procedure, potential risks (including facial paresis and masticatory weakness), and expected outcomes.

This retrospective case series included three patients with PMS who were classified as “DBS non‐responders.” DBS failure was defined based on the following criteria: (1) Clinical non‐response—less than 25% improvement in symptoms (assessed by BFMDRS or other clinical evaluation) or the emergence of intolerable stimulation‐induced adverse effects. (2) Optimization exhaustion—a minimum of 6 months of postoperative programming optimization, including adjustments to pulse width, frequency, voltage, and active contact configuration. (3) Ineligibility for revision—the patient was either confirmed to have optimal lead placement, or in cases of suspected suboptimal placement, was unwilling or unable to undergo surgical lead revision due to economic constraints, psychological stress, or medical contraindications.

### 2.2 Surgical Procedure

Procedures were performed under CT guidance using blunt radiofrequency needles targeting either the stylomastoid foramen (facial nerve) or foramen ovale (mandibular nerve). Target localization was verified via 3D reconstruction and positive motor stimulation (2 Hz, 0.3–1.0 mA).

For facial nerve ablation, the objective was to induce partial functional impairment to alleviate symptoms. The thermal dose was titrated to the clinical effect: XperCT 3D reconstruction showing DBS electrodes and the radiofrequency needle targeting the right stylomastoid foramen, starting at 65°C for 30 s, the temperature was increased in 5°C increments strictly until the clinical endpoint was observed. The procedure was terminated immediately upon the onset of slight air leakage during buccal distension or visible eyelashes on forced closure.

Mandibular nerve ablation utilized a similar symptom‐guided step‐heating protocol (starting at 65°C, in 5°C increments, 30 s per cycle). Temperature elevation was performed only if symptoms persisted, with the procedure targeting the cessation of dystonic jaw deviation and the development of facial hypesthesia in the mandibular distribution.

### 2.3 Case 1

**FIGURE 1:**
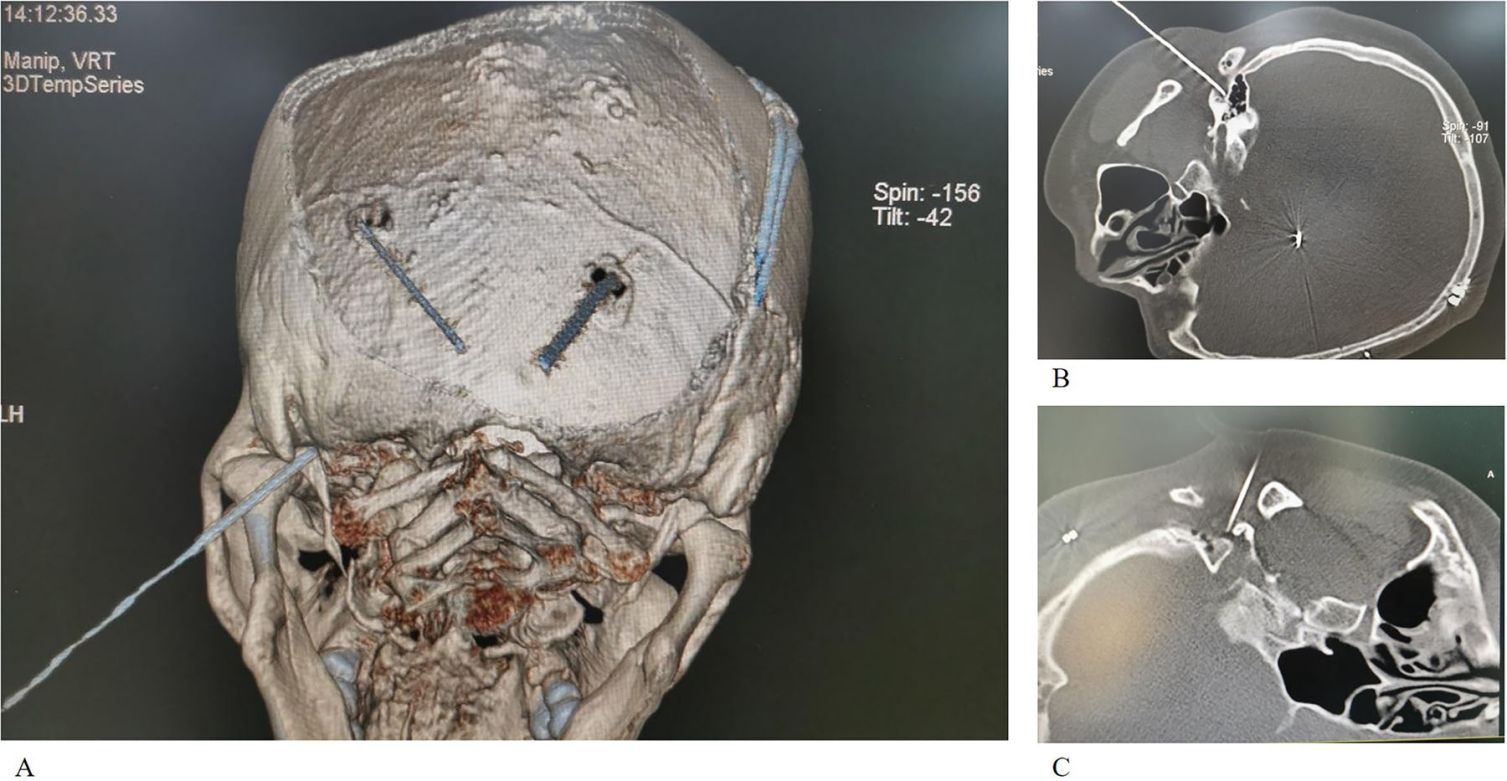
Intraoperative imaging of the first bilateral partial facial nerve radiofrequency ablation (RFA) in Case 1. (A) Three‐dimensional (3D) CT reconstruction showing the pre‐existing DBS electrodes and the radiofrequency needle targeting the left stylomastoid foramen. (B) CT‐guided needle placement at the left stylomastoid foramen. (C) CT‐guided needle placement at the right stylomastoid foramen.

**FIGURE 2:**
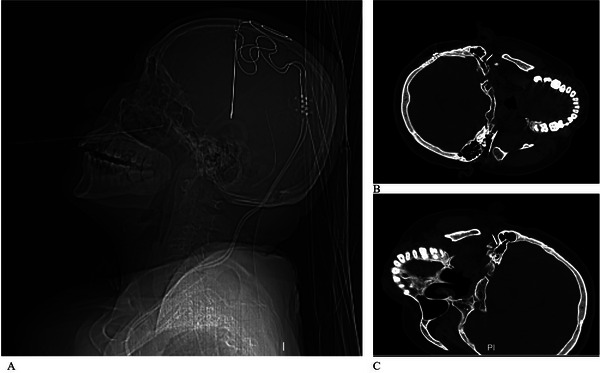
Intraoperative imaging of the repeat partial facial nerve RFA in Case 1. (A) CT 3D reconstruction visualizing the DBS electrodes and the radiofrequency needle trajectory toward the stylomastoid foramen. (B) CT‐guided needle placement at the right stylomastoid foramen. (C) CT‐guided needle placement at the left stylomastoid foramen.

A female in her early 60s presented with a 7‐year history of progressive, involuntary twitching of the eyelids and upper lip. The condition had no obvious inducement, initially manifesting as bilateral blepharospasm and gradually extending to involve the upper lip, exacerbated by emotional stress. Notably, no masticatory spasms were present. Review of systems was negative for constitutional symptoms or other neurological deficits. Despite multiple botulinum toxin injections, symptom relief was transient (2–4 months). In December 2021, she underwent bilateral DBS at an outside institution. Despite repeated postoperative programming adjustments, clinical improvement was marginal, and ongoing adjuvant botulinum toxin therapy remained necessary. She presented to our center in April 2023. Medical history and routine laboratory investigations were unremarkable. Cranial MRI revealed a vascular loop (left posterior inferior cerebellar artery) in proximity to the facial nerve root, but electromyography showed no abnormal muscle response (AMR), supporting the diagnosis of PMS. Following informed consent, bilateral facial nerve RFA was performed via the stylomastoid foramen (Li et al. 2025). The procedure was terminated immediately upon the resolution of spasms (Figure 1). Symptom recurrence occurred 14 months postoperatively, and a repeat bilateral partial RFA was successfully performed in June 2024 (Figure 2).

### 2.4 Case 2

**FIGURE 3:**
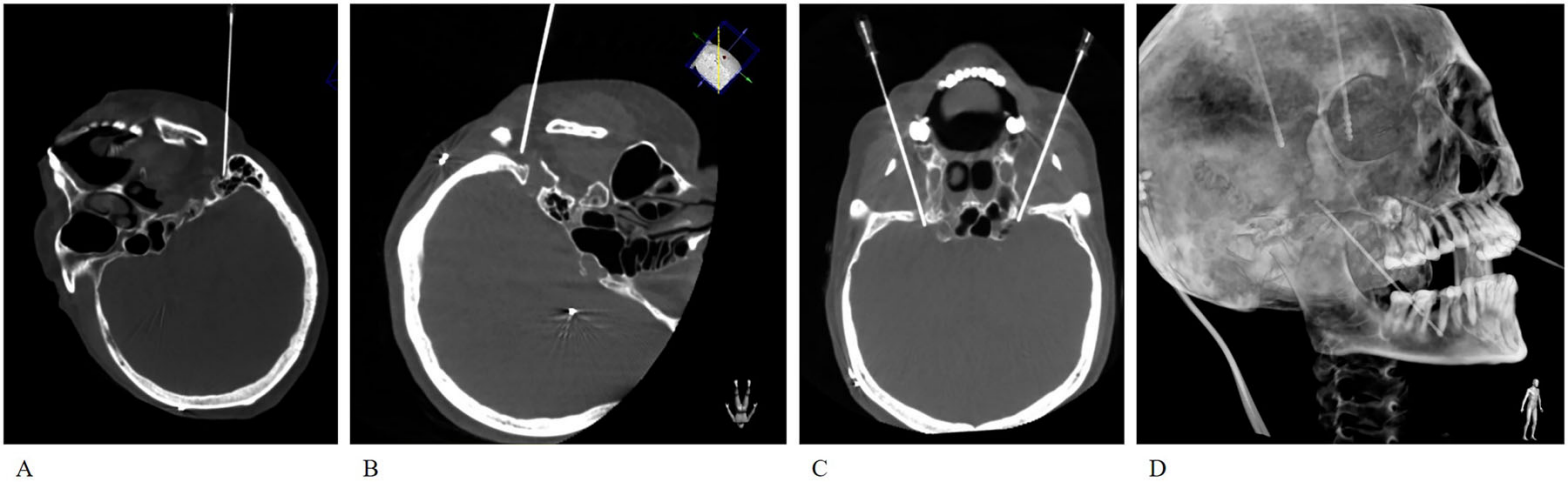
Combined extracranial RFA of the facial and mandibular nerves in Case 2. (A) Radiofrequency needle positioning at the left stylomastoid foramen. (B) Radiofrequency needle positioning at the right stylomastoid foramen. (C) Bilateral radiofrequency needle placement at the foramen ovale. (D) XperCT 3D reconstruction displaying DBS electrodes and radiofrequency needles targeting the bilateral foramen ovale.

A female in her late 50s presented with a 4‐year history of refractory blepharospasm and severe bruxism associated with trismus (difficulty opening the mouth). Pharmacotherapy and botulinum toxin injections had proven ineffective. In March 2022, she underwent DBS implantation at another facility. Despite extensive postoperative programming adjustments, her dystonic symptoms remained refractory. Furthermore, DBS activation induced stimulation‐dependent adverse effects, including bradyphrenia, dysarthria, gait initiation difficulties, and limb tremor. She visited our department in July 2024. Based on established protocols (H. Huang et al. 2024; Peng, Wang, et al. 2025), a combined extracranial RFA strategy was adopted. Under XperCT guidance, she underwent combined bilateral partial RFA of the facial nerve (stylomastoid foramen) and mandibular nerve (foramen ovale) (Figure 3) following the standard protocol described above. Immediate postoperative assessment showed complete remission of blepharospasm and bruxism. Sequelae were limited to mild facial paresis, hypesthesia in the mandibular distribution, and masticatory weakness.

### 2.5 Case 3

**FIGURE 4:**
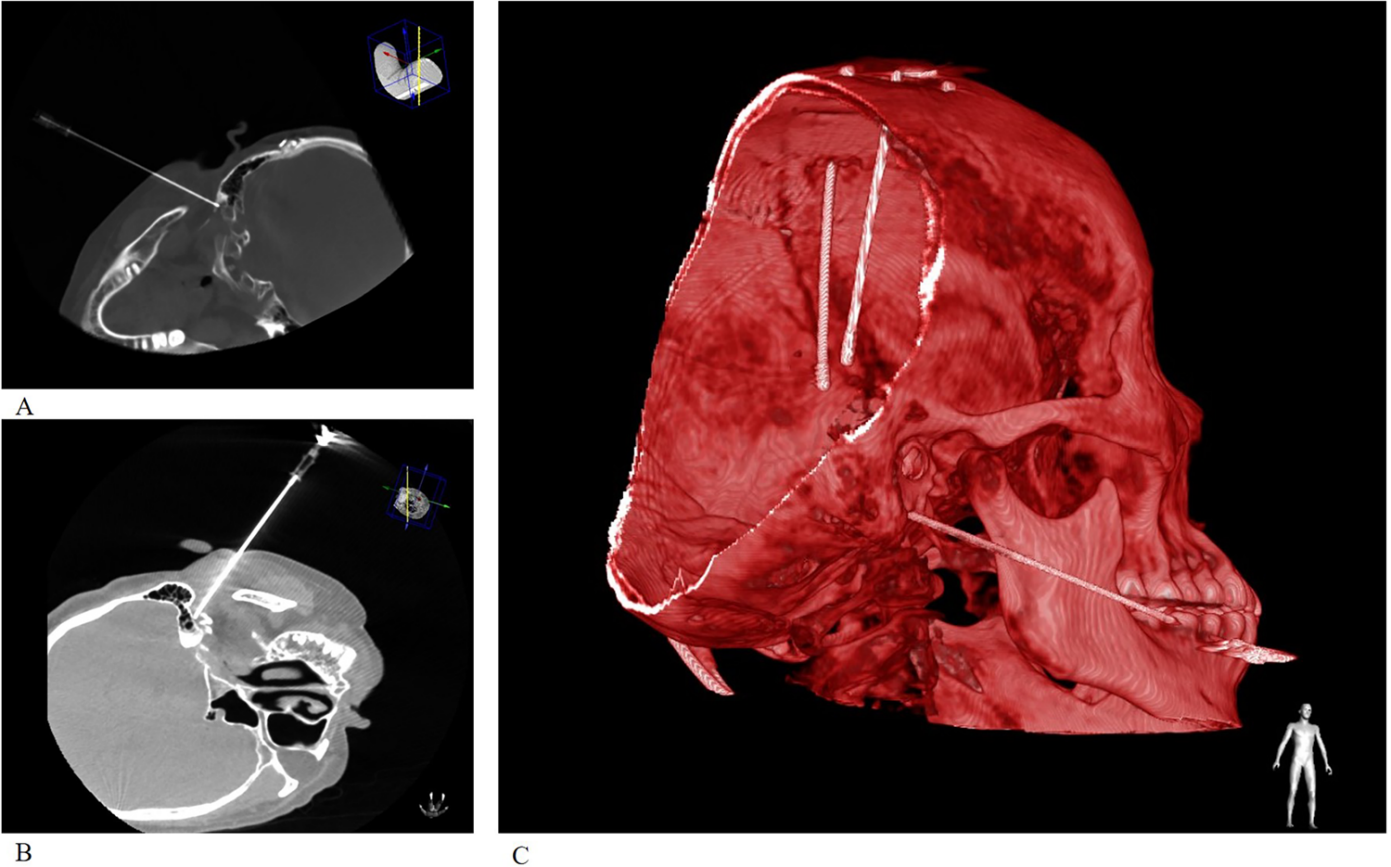
Bilateral partial facial nerve RFA in Case 3. (A) Radiofrequency needle advanced to the left stylomastoid foramen. (B) Radiofrequency needle placement at the right stylomastoid foramen. (C)XperCT 3D reconstruction showing DBS electrodes and the radiofrequency needle targeting the right stylomastoid foramen.

A female in her early 60s with a 24‐year history of PMS presented 10 years after DBS surgery. Her symptoms had remained suboptimal despite long‐term stimulation. In February 2025, she underwent XperCT‐guided bilateral partial facial nerve RFA targeting the stylomastoid foramen (Figure 4). The procedure resulted in the immediate cessation of blepharospasm and perioral twitching, with only mild residual facial paralysis.

## 3 Results

### 3.1 Case 1

Following the initial procedure, the patient achieved complete resolution of blepharospasm and oromandibular dystonia. The Burke–Fahn–Marsden Dystonia Rating Scale Movement subscale (BFMDRS‐M) score decreased from 9 to 0, and the Disability subscale (BFMDRS‐D) score improved from 2 to 1. Clinical remission was sustained for 14 months until symptom recurrence was noted (BFMDRS‐M 7.5, BFMDRS‐D 2). A second bilateral partial RFA of the facial nerve was performed in June 2024, resulting in immediate symptom cessation (Post‐operative BFMDRS‐M 0, BFMDRS‐D 1). At the 10‐month follow‐up, the patient remains symptom‐free with no signs of recurrence.

### 3.2 Case 2

Immediate postoperative assessment revealed significant alleviation of blepharospasm and bruxism. The BFMDRS‐M score dropped from 14 to 0.5, and the BFMDRS‐D score improved from 5 to 2. Procedural sequelae included mild facial paresis, mandibular hypesthesia, and mild masticatory weakness. Facial paresis resolved completely by the 3‐month follow‐up. By 6 months, sensory function in the mandibular distribution had normalized, and masticatory power had substantially recovered. At the 11‐month follow‐up, no recurrence of dystonic symptoms was observed.

### 3.3 Case 3

The patient experienced immediate relief of blepharospasm and perioral twitching post‐procedure. BFMDRS scores improved from a baseline of BFMDRS‐M 6 and BFMDRS‐D 1 to post‐operative scores of BFMDRS‐M 0 and BFMDRS‐D 0. Adverse effects were limited to mild facial paresis, which resolved spontaneously within 3 months. At the 6‐month follow‐up, the patient maintained a complete response with no recurrence.

## 4 Discussion

PMS represents a complex therapeutic challenge. While defined as a segmental craniocervical dystonia characterized by involuntary contractions of the orbicularis oculi and oromandibular musculature (Pandey and Sharma 2017), its pathophysiology remains incompletely understood despite being described over a century ago (Henry 1910). Current hypotheses implicate dysfunction in the basal ganglia–thalamus–cortex circuit, specifically involving neurotransmitter dysregulation and an imbalance between excitatory and inhibitory signaling (LeDoux 2009; Ma et al. 2021).

Diagnostically, PMS is distinct from hemifacial spasm based on the absence of AMR on electromyography (C. Huang et al. 2016; Defazio et al. 2021). However, management remains difficult. Pharmacotherapy (e.g., haloperidol, clonazepam) offers limited benefit. While botulinum toxin injections are the standard of care, efficacy is transient (approx. 6 months) and may diminish over time (Duarte et al. 2024; Jochim et al. 2020; Wu et al. 2024). DBS is currently regarded as the primary surgical option for refractory cases; yet, 30%–50% of patients fail to achieve satisfactory outcomes (Hao et al. 2024; Xie et al. 2025). This suboptimal efficacy may stem from the generalized modulation of the GPi or STN, which does not selectively target the specific somatotopic representations of the affected cranial nerves (facial, trigeminal, accessory nerves, etc.).

We propose that partial RFA serves as a distinct therapeutic modality by targeting the neural transmission pathway. Conceptually, current treatments target different levels of the motor circuit: DBS modulates the signal source (basal ganglia pacemaker). Botulinum toxin blocks the effector (neuromuscular junction). Partial RFA blocks the conduction pathway (cranial nerve trunk). By selectively blocking ectopic impulses along the facial or mandibular nerves, RFA prevents abnormal excitatory signals from reaching the effector muscles. This “intermediate link” blockade explains why RFA can be effective even when central modulation (DBS) fails. Building on our experience with hemifacial spasm (B. Huang et al. 2021) and blepharospasm (B. Huang et al. 2022), we extended this technique to DBS‐refractory PMS.

In this preliminary case series, three patients with DBS failure achieved symptom remission following partial RFA. These findings suggest that extracranial RFA may serve as a valuable salvage therapy for this specific population. Unlike DBS, which requires complex stereotactic planning, extracranial RFA is target‐specific. For instance, blepharospasm is addressed via the facial nerve, while oromandibular dystonia is managed via the mandibular nerve.

The critical technical challenge of this procedure lies in achieving a “therapeutic balance” between symptom suppression and functional preservation. Complete ablation is contraindicated as it results in permanent paralysis. Our protocol employs a graded thermal lesioning strategy to induce mild, controlled functional impairment. The clinical endpoint is defined by the onset of subtle functional deficits: Slight air leakage during buccal distension or visible eyelashes on forced closure (facial nerve), or subjective hypesthesia and decreased bite force (mandibular nerve). We acknowledge that current reliance on intraoperative clinical observation is somewhat subjective. Future studies should aim to incorporate objective electrophysiological monitoring—such as measuring decrements in nerve conduction velocity or compound muscle action potentials (CMAP)—to quantify the degree of ablation more precisely and minimize the risk of excessive nerve damage.

Given that the procedure involves partial lesioning, nerve regeneration and symptom recurrence are potential outcomes. Our previous data suggest a remission period of approximately 11–18 months (H. Huang et al. 2024; B. Huang et al. 2021; Lin et al. 2022). However, as demonstrated in Case 1, recurrence can be effectively managed with repeat ablation. Importantly, the minimally invasive nature of RFA makes repeated treatment acceptable to patients, particularly given its favorable safety profile and significantly lower cost (approximately 1/10th that of DBS) (Li et al. 2025; Peng, Li, et al. 2025).

This study is limited by its retrospective design and small sample size (*n* = 3). Consequently, these findings should be interpreted as exploratory. While the short‐ to medium‐term outcomes are encouraging, larger prospective cohorts with longer follow‐up are required to validate the safety and long‐term efficacy of this approach.

## 5 Conclusion

In conclusion, this preliminary case series suggests that partial RFA of the involved cranial nerves is a feasible and potentially effective salvage therapy for PMS patients who have failed DBS. By selectively interrupting the aberrant signal transmission along the peripheral nerve, this technique offers a distinct mechanism of action compared to central neuromodulation. While the procedure carries a risk of transient functional deficits, these sequelae were manageable and reversible in our cohort. Given its minimal invasiveness, cost‐effectiveness, and repeatability, extracranial RFA warrants further investigation as a complementary option in the therapeutic algorithm for refractory PMS. Future prospective studies with larger sample sizes are essential to validate these findings and to establish standardized electrophysiological protocols for optimizing the critical balance between therapeutic efficacy and nerve preservation.

## Author Contributions

**Xue Li**: conceptualization, data curation, funding acquisition, writing – original draft. **Min Yan**: funding acquisition, investigation, writing – review and editing. **Bing Huang**: funding acquisition, methodology, writing – original draft. **Lian Yu**: project administration, supervision, writing – review and editing. **Huabo Liu**: Funding acquisition, writing – review and editing. **Hao Huang**: data curation, writing – review and editing. **Ming Yao**: writing – review and editing.

## Funding

Zhejiang Provincial Medical and Health Science and Technology Plan(2025KY855, 2025KY1801), Zhejiang Clinovation Pride(CXTD202501020), The National Clinical Key Specialty Construction Project of China 2021(2021‐LCZDZK‐01), Leading Health Talents of Zhejiang Province, Zhejiang Health Office No. 18(2020), Zhejiang Key Laboratory of Pain Perception and Neuromodulation.

## Ethics Statement

The study protocol was approved by the institutional ethics committees of the participating hospitals (2022‐LY317, 2024–031). All enrolled patients provided comprehensive written informed consent, which included permission for the use of their clinical data and images for academic purposes.

## Data Availability

Data sharing is not applicable to this article as no new data were created or analyzed in this study.

## References

[brb371383-bib-0001] Defazio, G. , H. A. Jinnah , A. Berardelli , et al. 2021. “Diagnostic Criteria for Blepharospasm: A Multicenter International Study.” Parkinsonism & Related Disorders 91: 109–114. 10.1016/j.parkreldis.2021.09.004. 34583301 PMC9048224

[brb371383-bib-0002] Duarte, A. , L. Coutinho , F. M. B. Germiniani , and H. A. G. Teive . 2024. “Effects of Onabotulinum Toxin Type A Injections in Patients With Meige's Syndrome.” Arquivos de neuro‐psiquiatria 82: s00441785691. 10.1055/s-0044-1785691. 38641339 PMC11031253

[brb371383-bib-0004] Hao, Q. , W.‐T. Zheng , Z.‐H. Zhang , et al. 2024. “Subthalamic Nucleus Deep Brain Stimulation in Primary meige Syndrome: Motor and Non‐motor Outcomes.” European Journal of Neurology 31: e16121. 10.1111/ene.16121. 37933887 PMC11235968

[brb371383-bib-0005] Hassell, T. J. W. , and D. Charles . 2020. “Treatment of Blepharospasm and Oromandibular Dystonia With Botulinum Toxins.” Toxins 12: 269. 10.3390/toxins12040269. 32331272 PMC7232182

[brb371383-bib-0006] Henry, M. 1910. “Les Convulsions de la Face: Uneformeclinique de Convulsion Faciale,Bilaterale et Mediane.” Revue Neurologique 10: 437–443.

[brb371383-bib-0007] Huang, B. , X.‐D. Du , M. Yao , H.‐D. Lin , W.‐H. Yu , and Q.‐H. Zhou . 2022. “CT‐Guided Radiofrequency Ablation of the Extracranial Cranial Nerve for the Treatment of Meige's Syndrome.” Frontiers in Neuroscience 16: 1013555. 10.3389/fnins.2022.1013555. 36278012 PMC9582605

[brb371383-bib-0008] Huang, B. , M. Yao , Q. Chen , et al. 2021. “Awake CT‐Guided Percutaneous Stylomastoid Foramen Puncture and Radiofrequency Ablation of Facial Nerve for Treatment of Hemifacial Spasm.” Journal of Neurosurgery 135: 1459–1465. 10.3171/2020.10.JNS203209. 33862595

[brb371383-bib-0009] Huang, C. , S. Miao , H. Chu , et al. 2016. “Application of Electrophysiological Methods and Magnetic Resonance Tomographic Angiography in the Differentiation Between Hemifacial Spasm and Meige Syndrome.” Neurological Sciences 37: 769–775. 10.1007/s10072-016-2492-2. 26838523

[brb371383-bib-0010] Huang, H. , B. Huang , X. Du , et al. 2024. “CT‐Guided Radiofrequency Ablation of Facial and Mandibular Nerves in the Treatment of Compound Meige's Syndrome.” Neuroradiology 66: 1761–1764. 10.1007/s00234-024-03392-1. 38844696 PMC11424667

[brb371383-bib-0011] Jochim, A. , T. Meindl , C. Huber , et al. 2020. “Treatment of Blepharospasm and Meige's Syndrome With Abo‐ and Onabotulinumtoxina: Long‐Term Safety and Efficacy in Daily Clinical Practice.” Journal of Neurology 267: 267–275. 10.1007/s00415-019-09581-w. 31630241

[brb371383-bib-0012] LeDoux, M. S. 2009. “Meige Syndrome: What's in a Name?” Parkinsonism & Related Disorders 15: 483–489. 10.1016/j.parkreldis.2009.04.006. 19457699 PMC2743078

[brb371383-bib-0013] Li, X. , Y. Ma , H. Lin , et al. 2025. “Computed Tomography‐Navigated Radiofrequency Ablation for Meige's Syndrome: A Game‐Changer in Treatment.” Asian Journal of Surgery 48: 281–286. 10.1016/j.asjsur.2024.09.042. 39277463

[brb371383-bib-0014] Lin, H. , G. Cao , Z. Yang , et al. 2022. “Comparison of Two Puncture Approaches in CT‐Guided Percutaneous Radiofrequency Ablation at the Stylomastoid Foramen for Treatment of Hemifacial Spasm.” Pain Physician 25: E1063–E1071. 36288592

[brb371383-bib-0015] Ma, H. , J. Qu , L. Ye , Y. Shu , and Q. Qu . 2021. “Blepharospasm, Oromandibular Dystonia, and Meige Syndrome: Clinical and Genetic Update.” Frontiers in Neurology 12: 630221. 10.3389/fneur.2021.630221. 33854473 PMC8039296

[brb371383-bib-0016] Pandey, S. , and S. Sharma . 2017. “Meige's Syndrome: History, Epidemiology, Clinical Features, Pathogenesis and Treatment.” Journal of the Neurological Sciences 372: 162–170. 10.1016/j.jns.2016.11.053. 28017205

[brb371383-bib-0017] Peng, H. , X. Li , and B. Huang . 2025. “Management of Meige Syndrome With Bilateral Trigeminal and Facial Nerves Combing.” Frontiers in Neurology 16: 1626729. 10.3389/fneur.2025.1626729. 40800689 PMC12340520

[brb371383-bib-0018] Peng, H. , C. Wang , B. Xin , and B. Huang . 2025. “CT‐Guided Extracranial Radiofrequency of Multiple Groups of Cranial Nerves for the Treatment of Compound Meige's Syndrome.” Korean Journal of Pain 38: 209–212. 10.3344/kjp.24415. 40159940 PMC11965996

[brb371383-bib-0019] Wu, X. , T. Xue , S. Pan et al. 2024. “Pallidal Versus Subthalamic Deep Brain Stimulation for Meige Syndrome: A Systematic Review and Meta‐Analysis.” Heliyon 10: e27945. 10.1016/j.heliyon.2024.e27945. 38510025 PMC10950702

[brb371383-bib-0020] Xie, H. , J. Huang , Y. Diao , et al. 2025. “Efficacy Comparison and Outcome Predictors of GPi‐ and STN‐Targeted Deep Brain Stimulation for Meige Syndrome: A Systematic Review of Individual Patient Data.” Journal of Neurosurgery 142: 1577–1588. 10.3171/2024.9.JNS241263. 39854720

